# Pain profiles in pancreatic cancer and chronic pancreatitis: a multicenter cross-sectional analysis

**DOI:** 10.3389/fpain.2026.1903789

**Published:** 2026-09-11

**Authors:** Marie-Theres Kassik, Mahya Faghih, Conrad Bandhauer, Josefine Jäger, Sophie Rauschenberg, Søren S. Olesen, Daniel A. Laheru, Lei Zheng, Anna E. Phillips, Dhiraj Yadav, Jonas Rosendahl, Vikesh K. Singh, Asbjørn M. Drewes, Marko Damm

**Affiliations:** 1 Department of Internal Medicine I, University Hospital Halle, Martin-Luther-University Halle-Wittenberg, Halle (Saale), Germany; 2 Division of Gastroenterology, Department of Medicine, Johns Hopkins Medical Institutions, Baltimore, MD, United States; 3 Pancreatitis Center, Department of Medicine, Johns Hopkins Medical Institutions, Baltimore, MD, United States; 4 Centre for Pancreatic Diseases and Mech-Sense, Department of Gastroenterology and Hepatology, Aalborg University Hospital, Aalborg, Denmark; 5 The Sidney Kimmel Comprehensive Cancer Center, Johns Hopkins Medical Institutions, Baltimore, MD, United States; 6 Division of Gastroenterology, Hepatology, and Nutrition, University of Pittsburgh School of Medicine, Pittsburgh, PA, United States

**Keywords:** pain, pain management, pancreatic cancer, quality of life, sensory testing

## Abstract

**Introduction:**

Abnormal pain processing has been associated with impaired patient-reported outcomes (PROMs) in chronic pancreatitis (CP), but its clinical relevance in pancreatic ductal adenocarcinoma (PDAC) remains unclear. We investigated the association between P-QST-defined hyperalgesia and pain-related, psychological, and quality-of-life outcomes in patients with painful PDAC and compared these associations with CP.

**Methods:**

This multicenter cross-sectional analysis included patients with painful CP and PDAC at five tertiary referral hospitals in Denmark, Germany and the USA. Based on the results of pancreatic quantitative sensory testing (P-QST), patients were classified into three subgroups according to hyperalgesia status: no hyperalgesia, reflecting normal pain processing; segmental hyperalgesia at the pancreatic dermatome, suggesting abnormal spinal pain processing; widespread hyperalgesia, indicative of pathological pain processing across the entire neuraxis. PROMs included the Brief Pain Inventory (BPI), Pain Catastrophizing Scale (PCS), Hospital Anxiety and Depression Scale (HADS), and EORTC QLQ-C30. Between-disease P-QST analyses were adjusted for age, sex, and regular opioid use. Exploratory disease-by-hyperalgesia interaction analyses assessed whether associations between hyperalgesia and selected PROMs differed between CP and PDAC.

**Results:**

A total of 275 patients (153 CP, 122 PDAC) were enrolled. Hyperalgesia was present in 53.6% of patients with CP and 43.8% of patients with PDAC, with no statistically significant difference after multivariable adjustment (aOR 0.75, 95% CI 0.43–1.33; *p* = 0.327). Patients with CP reported greater pain intensity (*p* = 0.027), pain interference (*p* = 0.049), and pain catastrophizing (*p* = 0.034) than patients with PDAC. Within the PDAC cohort, hyperalgesia was not associated with consistent differences in pain intensity, pain interference, catastrophizing, anxiety, or quality-of-life outcomes. However, formal interaction analysis demonstrated that the association between hyperalgesia and BPI pain interference differed between PDAC and CP (interaction *β*=1.61, 95% CI 0.27–2.95; *p* = 0.018).

**Conclusion:**

Although altered pain processing was frequent in painful PDAC, its association with patient-reported outcomes differed from that observed in CP. In particular, hyperalgesia showed a disease-specific relationship with pain-related functional impairment. These findings suggest that the clinical meaning of P-QST-defined hyperalgesia in PDAC cannot simply be inferred from CP and support further PDAC-specific studies linking pain phenotype to patient-reported burden and treatment response and pain management approaches might not be transferable across diseases.

## Introduction

Pancreatic ductal adenocarcinoma (PDAC) is one of the cancer types with the poorest prognosis worldwide, exhibiting a mortality-to-incidence ratio of approximately 95% (1, 2). Alongside numerous diagnostic and therapeutic challenges, improving the quality of life for affected patients plays a major role. Many patients with PDAC already suffer from significant symptoms at the time of diagnosis. The most prominent of these is pain, which occurs in about 80% over the course of the disease (3, 4).

Inadequately treated pain not only leads to physical limitations but also negatively impacts various aspects of quality of life (5–7) Many patients suffer from anxiety, mood swings and emotional distress (8). Cancer-related pain is also frequently associated with depression, and its co-occurrence has additive negative effects on quality of life (9). In the context of PDAC, where life expectancy is often very limited, and the symptom burden is high, the future integration of palliative care and pain management expertise into patient care appears to be important (10).

The underlying mechanisms of pain generation and processing in PDAC have not yet been fully elucidated. To gain a better understanding of this pathophysiology and to establish personalized approaches for targeted pain management, pancreas-specific quantitative sensory testing (P-QST) was developed. It enables the characterization of pain processing in peripheral and central nociceptive pathways. Using P-QST patients with chronic pancreatitis (CP) can be assigned to three distinct pain phenotypes: normal pain processing, segmental hyperalgesia (hypersensitivity predominantly in the region of the pancreatic dermatome), and widespread hyperalgesia (additional hypersensitivity in other dermatomes together with other features of abnormal pain processing) (11). As such P-QST proofed to be a feasible method for assessing pain processing in CP patients (12). Furthermore, it has been demonstrated that abnormal central pain processing in CP patients is associated with patient-reported outcome measures (PROMs), including the presence of depression, anxiety, and reduced quality of life. Recently published data have also shown such abnormal central pain processing in patients with painful PDAC (13).

The study hypothesizes that patients with painful CP and PDAC with hyperalgesia experience greater limitations according to PROMs. The current study aims to verify whether this is the case and to what extent. Thus, the identification of abnormal pain processing in patients with PDAC would have implications extending beyond pain management alone.

## Methods

### Study design and patient selection

This study was a multicenter cross-sectional analysis of prospectively enrolled cohorts of patients with painful chronic pancreatitis (CP) and pancreatic ductal adenocarcinoma (PDAC). Patients with CP were enrolled between January 2018 and July 2025 at four tertiary referral centers in the United States and Denmark. Patients with PDAC were enrolled at Johns Hopkins University Medical Center, Baltimore, USA, and Martin Luther University Halle-Wittenberg, Halle (Saale), Germany, between January 2022 and July 2025. Patients were prospectively recruited using a convenience-based approach. Eligible patients identified at the participating centers were included.

The cohorts used in the present study partly originate from previously published P-QST studies. All 153 patients with CP included in the present analysis were part of a previously reported multicenter CP cohort comprising 201 patients (14). Of the 122 patients with PDAC included in the present study, 104 were included in our previous publication describing P-QST parameters and pain phenotypes in PDAC compared with healthy controls (13). The healthy control group was ≥18 years of age and had been characterized and described previously (12). Ethical approval was obtained for all participating cohorts and centers. The CP cohort was recruited under approvals from the University of Pittsburgh (IRB PRO17060648), Johns Hopkins University (IRB 00143375), Indiana University (IRB 1909843967), and Aalborg University Hospital (N-20090008). The healthy reference cohort was recruited under approvals from the University of Pittsburgh (IRB PRO17060648), Johns Hopkins University (IRB 00143375), and Aalborg University Hospital (N-20090008). The PDAC cohort was recruited under approvals from Johns Hopkins University (IRB 00143375) and Martin Luther University Halle-Wittenberg (IRB 2021-189). These approvals and the corresponding informed-consent procedures covered the present secondary analyses of the prospectively collected data.

Patients with CP were eligible if they fulfilled the criteria for definite CP according to the M-ANNHEIM classification. Patients with histologically confirmed PDAC were eligible if they were untreated or minimally treated at the time of study assessment. Up to four cycles of chemotherapy before enrolment were permitted. All patients reported pancreatic pain at the time of study assessment. Exclusion criteria were age <18 years, pregnancy, acute pancreatitis, or other painful conditions that could not reliably be distinguished from CP- or PDAC-related pain. No formal sample-size calculation was performed and all eligible participants available during the predefined recruitment period were included.

### Pain assessment

Based on the results of pancreatic quantitative sensory testing (P-QST), patients were classified into three subgroups according to hyperalgesia status: no hyperalgesia, segmental hyperalgesia or widespread hyperalgesia. The P-QST method and algorithm that leads to the distinction of different pain phenotypes has previously been published (11). By using pinpricks and applying pressure or a cold stimulus to pancreatic-specific and non-specific dermatomes, it is assessed whether increased afferent barrage from the pancreas may result in central sensitization exists due to convergence between afferents from pancreas and those of the skin in Th10 dermatome. Pressure pain detection (PPD) refers to the pressure at which the stimulus is perceived as painful. Pressure pain threshold (PPT) refers to the pressure at which the pain stimulus becomes intolerable. The index calculates the ratio of the values for pancreas-specific dermatome (Th10) to those for non-pancreas-specific dermatomes (C5, L1, L4). In summary, patients without hyperalgesia had normal pain sensation in selected dermatomes. Patients with segmental hyperalgesia had decreased pressure pain thresholds and/or abnormal temporal summation, tested by repetitive pinpricks, at the pancreatic “viscerotome” (Th10 dermatome). Temporal summation is the human proxy of the so-called “wind-up phenomenon” reflecting neuronal hyperexcitability. Widespread hyperalgesia denotes two or more of the following: decreased pressure pain thresholds, enhanced temporal summation at the forearm, increased cold pressor pain sensitivity, and impaired measures of descending inhibition from the brain to the spinal cord. For analyses comparing patients with and without hyperalgesia, segmental and widespread hyperalgesia were combined into a single hyperalgesia category. Regular opioid use was defined as the daily use of opioid medication as part of the patient's chronic medication regimen at the time of study assessment.

Clinical pain characteristics and Quality of Life (QoL) data (summarized as PROMs) were assessed using the Brief Pain Inventory short form (BPI-sf), the European Organization for Research and Treatment of Cancer Quality of Life Questionnaire Core 30 (EORTC-QLQ-C30), as well as the Hospital Anxiety and Depression Scale (HADS) and the Pain Catastrophizing Scale (PCS).

### Data analysis

Continuous variables are presented as mean ± standard deviation (SD), and categorical variables as *n* (%), unless otherwise specified. Unadjusted comparisons of continuous or ordinal variables between two groups were performed using the Mann–Whitney U test, and categorical variables were compared using the Pearson *χ*^2^ test.

The principal between-disease analyses of P-QST parameters were performed using multivariable linear regression for continuous outcomes. Pain phenotype was analyzed using multinomial logistic regression with normal pain phenotype as the reference category, and the presence of any hyperalgesia was analyzed using binary logistic regression. To apply a consistent adjustment strategy to the principal analyses, all models were adjusted for age, sex, and regular opioid use. CP served as the reference disease group. Results are reported as adjusted regression coefficients (*β*) or adjusted odds ratios (aOR), with 95% confidence intervals (CI).

Comparisons of PROMs between CP and PDAC, between PDAC patients with and without hyperalgesia, and between CP patients with and without hyperalgesia were considered exploratory.

To formally assess whether the association between hyperalgesia and PROMs differed according to underlying pancreatic disease, exploratory disease-by-hyperalgesia interaction analyses were performed for a limited set of clinically relevant outcomes representing pain intensity (BPI mean), pain interference (BPI interference), pain catastrophizing (PCS), depression, anxiety, and global quality of life (EORTC QLQ-C30 global QoL). These multivariable linear regression models included disease group, hyperalgesia status, the disease-by-hyperalgesia interaction term, age, sex, and regular opioid use. Interaction effects are reported as *β* coefficients with 95% CI.

Missing observations were not imputed. Analyses were performed using available cases for the variables included in each respective analysis; therefore, effective sample sizes varied between outcomes. The number of missing observations and/or effective denominators are reported in the corresponding tables.

All tests were two-sided, and *p* < 0.05 was considered statistically significant. Statistical analyses were performed using jamovi (version 2.6; The jamovi project, 2024).

## Results

### Patient characteristics

A total of 275 patients were prospectively enrolled from the five pancreatic referral centers. Of these patients, 153 had CP and 122 had histologically confirmed PDAC.

Patient characteristics are shown in Table 1. The mean age was 52 ± 13 years in the CP cohort and 66 ± 10 years in the PDAC cohort (*p* < 0.001). Women accounted for 67/153 (43.8%) of the CP cohort and 56/122 (45.9%) of the PDAC cohort. The racial composition differed between cohorts: 16/153 (10.5%) CP patients and 47/122 (38.5%) PDAC patients were recorded as Black. Among the 153 patients with chronic pancreatitis, the underlying etiologies were toxic-metabolic (alcohol-related) in 74 patients (48.4%), idiopathic in 43 (28.1%), genetic in 20 (13.1%), and other causes in 16 (10.5%). In the group of patients with alcohol-related chronic pancreatitis, 29 (39.2%) reported ongoing alcohol consumption, whereas the remaining patients had discontinued alcohol use.

**Table 1:** Baseline characteristics of patients with chronic pancreatitis (CP) and pancreatic ductal adenocarcinoma (PDAC).

Characteristic	CP *n* = 153	PDAC *n* = 122	*p-*values
Sex			*0*.*727*
Male	86 (56%)	66 (54%)	
Female	67 (44%)	56 (46%)	
Age			*<0*.*001*
Mean (SD)	52 (13)	66 (10)	
Ethnicity			*<0*.*001*
Black	16 (10%)	47 (39%)	
White	130 (85%)	74 (60%)	
Other	7 (5%)	1 (1%)	
Use of opioids			*<0*.*001*
no	60 (39%)	74 (61%)	
yes missings	92 (60%) 1 (1%)	48 (39%)	
Pancreatic exocrine insufficiency			*<0*.*001*
no	52 (34%)	91 (75%)	
yes missings	99 (65%) 2 (1%)	31 (25%)	
Diabetes mellitus			*0*.*636*
no	91 (50%)	69 (57%)	
yes missings	61 (40%) 1 (1%)	52 (43%) 1 (1%)	
Disease duration (months)	74.4 (74)	1.7 (2.7)	*<0*.*001*

Data are presented as mean ± standard deviation (SD) or *n* (%).

The majority of PDAC patients (80 patients, 66%) already had metastases at the time of enrollment. To minimize potential treatment-related effects on pain perception, the study preferentially included treatment-naïve PDAC patients, allowing a maximum of four chemotherapy cycles. Consequently, only 10 of the 122 PDAC patients (8.2%) had received chemotherapy before study inclusion. Three patients had received palliative chemotherapy (two patients with 1–2 cycles of FOLFIRINOX and one patient with one cycle of gemcitabine plus nab-paclitaxel). Seven patients had received neoadjuvant FOLFIRINOX (four with one cycle, two with two cycles, and one with three cycles).

The proportion of patients with regular opioid use was more frequent in CP than in PDAC. Among participants with available medication data, 92/152 (60.5%) CP patients and 48/122 (39.3%) PDAC patients regularly used opioids (*p* < 0.001). Besides opioids according to the WHO analgesic ladder, 79 PDAC patients received WHO step I analgesics. Thirty-six patients received adjuvant medications, including SSRIs/SNRIs (*n* = 11), benzodiazepines (*n* = 11), tricyclic antidepressants (*n* = 1), other antidepressants (*n* = 3), gabapentinoids/anticonvulsants (*n* = 12), corticosteroids (*n* = 11), and Z-drugs (*n* = 2). No cannabinoid use was documented in the available medication data. Information on illicit substance use, acupuncture, and other complementary pain-management approaches was not systematically collected. Intravenous opioid therapy was documented in one patient only, and no patient underwent interventional pain procedures such as celiac plexus neurolysis.

In addition, a higher proportion of CP patients had pancreatic exocrine insufficiency compared to PDAC patients (66% vs. 25%, *p* < 0.001), whereas the rate of endocrine insufficiency was similar (40% vs. 44%, *p* = 0.636).

### Pain phenotypes

The adjusted comparison of P-QST parameters and pain phenotypes is shown in Table 2. All principal models were adjusted for age, sex, and regular opioid use.

**Table 2:** Quantitative sensory testing (P-QST) parameters and pain phenotypes in patients with chronic pancreatitis (CP) and pancreatic ductal adenocarcinoma (PDAC).

P-QST parameter/pain phenotype	CP *n* = 153	PDAC *n* = 122	*Regression coefficient β* ^1^/ aOR^2^	*p-*values
Cold pressure endurance time in sec missings		1		
Mean	74	79	12.90 (95%CI 1.24–24.57)**^1^**	0.03
Standard deviation	44	41		
Conditioned pain modulation in % missings		1		
Mean	21.19	17.54	−3.19 (95%CI −11.38–5.00)^1^	0.444
Standard Deviation	26.97	30.88		
Sum pressure pain detection threshold, kPa				
Mean	1687	1532	−147.17 (95%CI −360.4–66.06)^1^	0.175
Standard Deviation	930	606		
Index of pressure pain detection threshold				
Mean	0.960	1.041	0.03 (95%CI −0.044–0.104)^1^	0.427
Standard Deviation	0.259	0.266		
Repetitive pinprick, right forearm, VAS				
Mean	3	3	−0.086 (95%CI −0.718–0.547)^1^	0.790
Standard Deviation	2	2		
Repetitive pinprick, TH10 abdomen, VAS				
Mean	3	3	−0.108 (95%CI −0.854–0.637)^1^	0.775
Standard Deviation	3	2		
Pain phenotype				
normal	71 (46.41)	68 (55.74)		ref.
segmental	46 (30.07)	27 (22.95)	0.81 (95%CI 0.41–1.62)^2^	0.550
widespread Not classified	36 (23.53)	26 (21.31) 1	0.69 (95%CI 0.34–1.41)^2^	0.304
Hyperalgesia				
no	71 (46.41)	68 (56.20)		ref.
yes Not classified	82 (53.59)	53 (43.80) 1	0.75 (95%CI 0.43–1.33)^2^	0.327

Data are presented as mean ± standard deviation (SD) or *n* (%). Linear regression analyses^1^ were performed for continuous outcomes and logistic regression analyses^2^ for categorical outcomes. Results are reported as regression coefficients (*β*) for linear models and adjusted odds ratios (aOR) with 95% confidence intervals (CI) for logistic models. CP served as the reference group. A *p*-value < 0.05 was considered statistically significant.

Cold pressor endurance was longer in patients with PDAC than in patients with CP, with an adjusted between-group difference of approximately 12.9 s (95% CI 1.2 to 24.6; *p* = 0.03). No statistically significant between-group differences were detected for conditioned pain modulation (adjusted *β* −3.19 percentage points, 95% CI −11.38 to 5.00; *p* = 0.444), sum pressure pain detection threshold (*β* −147.17 kPa, 95% CI −360.40 to 66.06; *p* = 0.175), pressure pain detection threshold index (*β* 0.030, 95% CI −0.044 to 0.104; *p* = 0.427), repetitive pinprick at the right forearm (*β* −0.086, 95% CI −0.718 to 0.547; *p* = 0.790), or repetitive pinprick at the TH10 abdomen (*β* −0.108, 95% CI −0.854 to 0.637; *p* = 0.775).

Pain phenotype could be classified in all 153 CP patients and in 121 of 122 PDAC patients. Among patients with CP, 71 (46.4%) had a normal pain phenotype, 46 (30.1%) segmental hyperalgesia, and 36 (23.5%) widespread hyperalgesia. Among evaluable patients with PDAC, 68/121 (56.2%) had a normal pain phenotype, 27/121 (22.3%) segmental hyperalgesia, and 26/121 (21.5%) widespread hyperalgesia.

After adjustment for age, sex, and regular opioid use, no statistically significant difference between PDAC and CP was detected for segmental hyperalgesia versus normal pain phenotype (aOR 0.81, 95% CI 0.41–1.62; *p* = 0.550) or widespread hyperalgesia versus normal pain phenotype (aOR 0.69, 95% CI 0.34–1.41; *p* = 0.304). When segmental and widespread hyperalgesia were combined, 82/153 (53.6%) CP patients and 53/121 (43.8%) PDAC patients had hyperalgesia. The adjusted odds of hyperalgesia did not differ statistically significantly between PDAC and CP (aOR 0.75, 95% CI 0.43–1.33; *p* = 0.327).

**Figure 1:**
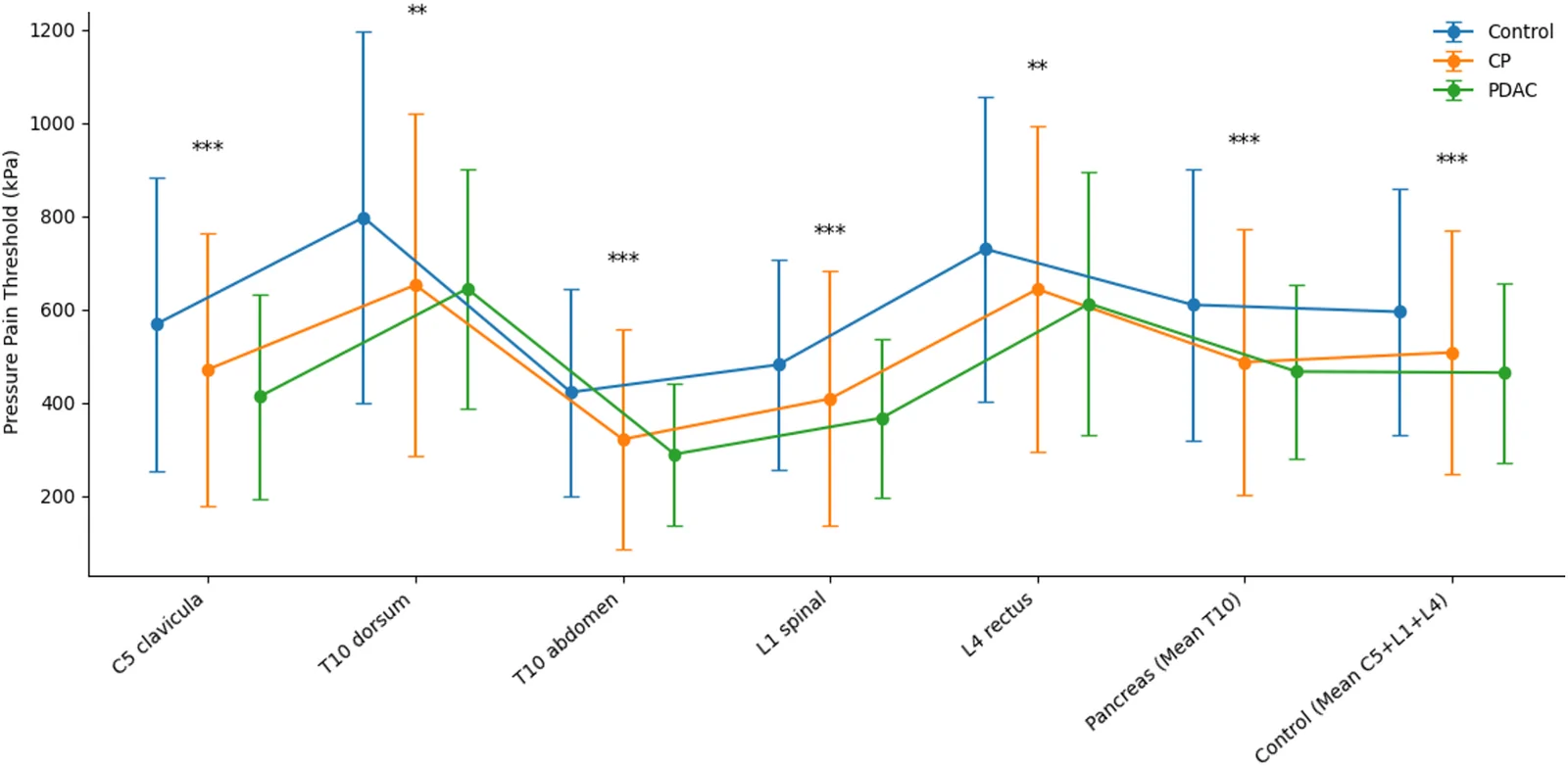
Comparison of pressure pain thresholds across groups. Pressure pain thresholds (PPT, kPa) at different anatomical sites are shown for healthy controls (Controls, *n* = 122), patients with chronic pancreatitis (CP, *n* = 153) and pancreatic adenocarcinoma (PDAC, *n* = 122). Data are presented as mean ± standard deviation. Group differences were analyzed using the Kruskal–Wallis test. Significance levels are indicated as follows: *p < 0.05, **p < 0.01, ***p < 0.001.

Pressure pain thresholds across individual anatomical sites in patients with CP and PDAC and in the previously characterized healthy reference cohort are displayed in Figure 1. Overall differences among the three groups were detected across the assessed sites using Kruskal–Wallis tests, with the corresponding significance levels shown in the figure. These analyses were descriptive across the three groups and were separate from the adjusted CP-versus-PDAC analyses reported in Table 2**.**

### Patient reported outcome measures (PROMs)

Exploratory comparisons of PROMs between CP and PDAC are presented in Table 3. Effective sample sizes varied between outcomes because of missing questionnaire data.

**Table 3:** Patient-reported outcome measures (PROMs) in CP patients vs. PDAC patients.

PROM	CP (153)	PDAC (122)	*p-*values
BPI mean missings	4		0.027^2^
Mean (SD)	4.2 (3)	3.6 (2)	
BPI relief missings	15	22	<0.001^2^
Mean (SD)	41.8 (31)	57.8 (34)	
BPI severe missings	4		0.003^1^
no	61 (41%)	72 (59%)	
yes	88 (59%)	50 (41%)	
BPI interference mean missings	4		0.049^2^
Mean (SD)	4.1 (3)	3.4 (3)	
PCS total missings	10		0.034^2^
Mean (SD)	23.3 (13)	20.1 (13)	
Depression score missings	10		0.563^2^
Mean (SD)	7 (4)	7 (5)	
Depression score > 7 missings	10		0.65^1^
No	79 (55%)	64 (53%)	
Yes	64 (45%)	58 (48%)	
Depression score ≥ 11 missings	10		0.727^1^
No	115 (80%)	96 (79%)	
Yes	28 (20%)	26 (21%)	
Anxiety score missings	10		0.548^2^
Mean	8 (5)	8 (4)	
Anxiety score > 7 missings	10		0.908^1^
No	69 (48%)	58 (48%)	
Yes	74 (52%)	64 (53%)	
Anxiety score ≥ 11 missings	10		0.601^1^
No	105 (73%)	93 (76%)	
Yes	38 (26%)	29 (24%)	
EORTC physical functioning missings	2		0.103^2^
Mean (SD)	72.2 (21)	66.3 (26)	
EORTC role functioning missings	2		0.816^2^
Mean (SD)	52.4 (34)	52.7 (38)	
EORTC emotional functioning missings	1		0.363^2^
Mean (SD)	61.9 (28)	58.7 (28)	
EORTC cognitive functioning missings	1		0.108^2^
Mean (SD)	67.3 (28)	72.5 (28)	
EORTC social functioning missings	3	1	0.503^2^
Mean (SD)	57.7 (34)	59.8 (35)	
EORTC quality of life missings	4	1	0.423^2^
Mean (SD)	46.4 (25)	43.8 (24)	
EORTC general symptoms missings	3	1	0.723^2^
Mean (SD)	41.8 (22)	41.7 (25)	
EORTC fatigue Missings	1		0.76^2^
Mean (SD)	53.3 (27)	52.1 (29)	
EORTC nausea and vomiting missings	1		0.072^2^
Mean (SD)	29.4 (31)	23.5 (31)	
EORTC pain missings	1		0.242^2^
Mean (SD)	60.6 (33)	56.1 (32)	
EORTC dyspnea missings	1		0.864^2^
Mean (SD)	19.5 (28)	20.8 (29)	
EORTC insomnia missings	1		0.509^2^
Mean (SD)	50.7 (38)	47.8 (41)	
EORTC appetite loss missings	1		0.683^2^
Mean (SD)	43.2 (35)	42.1 (40)	
EORTC constipation missings	1		0.836^2^
Mean (SD)	21.9 (30)	24 (35)	
EORTC diarrhea missings	1		0.244^2^
Mean (SD)	27.0 (31)	26.0 (37)	
EORTC financial difficulties missings	3	1	<0.001^2^
Mean (SD)	44.2 (39)	22.0 (34)	

Data are presented as mean ± SD or *n* (%). Categorical variables were compared using the pearson *χ*^2^ test^1^ and continuous variables using the Mann–Whitney U test^2^. Depression and anxiety are shown as continuous scores and by established cut-offs (>7 and >11). BPI mean represents average pain intensity (0–10). *BPI severe pain (mean 4–10)* indicates patients with a mean pain score ≥4 (moderate to severe pain). BPI relief reflects percentage pain reduction, and pain interference describes pain-related functional impairment. EORTC QLQ-C30 scales range from 0 to 100; higher scores indicate better functioning (function scales) or greater symptom burden (symptom scales). .

CP, chronic pancreatitis; PDAC, pancreatic ductal adenocarcinoma; SD, standard deviation; BPI, brief pain inventory; PCS, pain catastrophizing scale.

Patients with CP reported greater pain intensity than patients with PDAC (Brief pain inventory (BPI) mean 4.22 ± 2.57 vs. 3.58 ± 2.08; *p* = 0.027), and a greater proportion had moderate-to-severe pain (59.1% vs. 41.0%; *p* = 0.003). CP patients also reported less pain relief (41.81 ± 31.09% vs. 57.8 ± 34%; *p* < 0.001) and greater pain interference (4.08 ± 2.88 vs. 3.39 ± 2.70; *p* = 0.049).

Pain Catastrophizing Scale (PCS) scores were higher in CP than PDAC (23.29 ± 13.43 vs. 20.08 ± 12.49; *p* = 0.034). In contrast, continuous depression and anxiety scores and the proportions exceeding the Hospital Anxiety and Depression Scale (HADS) thresholds of >7 or ≥11 did not differ statistically significantly between disease groups.

Among the EORTC Quality of Life Core Questionnaire (EORTC QLQ-C30) outcomes, financial difficulties were greater in CP than PDAC (44.22 ± 39.10 vs. 22.04 ± 34.04; *p* < 0.001). No other EORTC QLQ-C30 domain met the nominal *p* < 0.05 threshold.

Table 4 shows a comparison of PROMs between 121 PDAC patients including 53 patients with ‘hyperalgesia’ (combining segmental and widespread hyperalgesia) and 68 patients with a normal pain phenotype (‘no hyperalgesia’). There were no statistically significant differences between these groups in BPI mean pain intensity (3.39 ± 2.22 vs. 3.68 ± 1.96; *p* = 0.384), BPI pain relief (56.07 ± 35.67% vs. 59.65 ± 31.90%; *p* = 0.658), pain interference (2.95 ± 2.23 vs. 3.70 ± 3.00; *p* = 0.257), or PCS total score (21.21 ± 12.47 vs. 19.04 ± 12.55; *p* = 0.424).

**Table 4:** Patient-reported outcome measures (PROMs) in PDAC patients stratified by hyperalgesia.

PROM	No hyperalgesia (*n* = 68)	Hyperalgesia (*n* = 53)	*p-*values
BPI mean			0.384^2^
Mean (SD)	3.7 (2)	3.4 (2)	
Opioid use			0.715^1^
no	42 (62%)	32 (59%)	
yes	26 (38%)	22 (41%)	
BPI relief missings	11	11	0.658^2^
Mean (SD)	59.7 (32)	56.1 (36)	
BPI severe pain			0.841^1^
no	41 (60%)	31 (58%)	
yes	27 (40%)	22 (42%)	
BPI interference mean			0.257^2^
Mean (SD)	3.7 (3)	2.9 (2)	
PCS total			0.424^2^
Mean (SD)	19 (13)	21.2 (13)	
Depression score			0.242^2^
Mean (SD)	7 (4)	8 (5)	
Depression score > 7			0.040^1^
No	41 (60%)	22 (42%)	
Yes	27 (40%)	31 (58%)	
Depression score ≥11			0.244^1^
No	56 (82%)	42 (80%)	
Yes	12 (18%)	11 (20%)	
Anxiety score			0.126^2^
Mean (SD)	8 (4)	9 (4)	
Anxiety score > 7			0.068^1^
No	37 (54%)	20 (38%)	
Yes	31 (46%)	33 (62%)	
Anxiety score ≥ 11			0.324^1^
No	54 (79%)	38 (72%)	
Yes	14 (21%)	15 (28%)	
EORTC physical functioning			0.721^2^
Mean (SD)	66.3 (27)	65.8 (24)	
EORTC role functioning			0.343^2^
Mean (SD)	49.3 (39)	56.3 (37)	
EORTC emotional functioning			0.985^2^
Mean (SD)	58.8 (28)	58.3 (27)	
EORTC cognitive functioning			0.378^2^
Mean (SD)	74.3 (27)	69.8 (29)	
EORTC social functioning missings	1		0.108^2^
Mean (SD)	54.7 (36)	65.4 (33)	
EORTC quality of life missings	1		0.720^2^
Mean (SD)	43.3 (23)	45.0 (25)	
EORTC general symptoms missings	1		0.398^2^
Mean (SD)	39.7 (23)	44.6 (27)	
EORTC fatigue			0.918^2^
Mean (SD)	52.8 (29)	52 (29)	
EORTC nausea and vomiting			0.835^2^
Mean (SD)	22.8 (31)	24.8 (32)	
EORTC pain			0.557^2^
Mean (SD)	54.7 (33)	58.2 (32)	
EORTC dyspnea			0.680^2^
Mean (SD)	21.1 (27)	20.8 (32)	
EORTC insomnia			0.625^2^
Mean (SD)	46.6 (40)	50.3 (43)	
EORTC appetite loss			0.305^2^
Mean (SD)	39.2 (40)	46.5 (39)	
EORTC constipation			0.505^2^
Mean (SD)	21.6 (32)	27.7 (39)	
EORTC diarrhea			0.833^2^
Mean (SD)	26 (38)	26.4 (36)	
EORTC financial difficulties missings	1		0.750^2^
Mean (SD)	20.4 (32)	24.5 (37)	

Data are presented as mean ± SD or *n* (%). Categorical variables were compared using the pearson *χ*^2^ test^1^ and continuous variables using the Mann–Whitney U test^2^. Depression and anxiety are shown as continuous scores and by established cut-offs (>7 and >11). BPI mean represents average pain intensity (0–10). *BPI severe pain (mean 4–10)* indicates patients with a mean pain score ≥4 (moderate to severe pain). BPI relief reflects percentage pain reduction, and pain interference describes pain-related functional impairment. EORTC QLQ-C30 scales range from 0 to 100; higher scores indicate better functioning (function scales) or greater symptom burden (symptom scales).

CP, chronic pancreatitis; PDAC, pancreatic ductal adenocarcinoma; SD, standard deviation; BPI, brief pain inventory; PCS, pain catastrophizing scale.

The continuous HADS depression and anxiety scores also did not differ statistically significantly according to hyperalgesia status. A HADS depression score >7 was observed in 31/53 (58.5%) patients with hyperalgesia and 27/68 (39.7%) patients with a normal pain phenotype (*p* = 0.040). However, the corresponding comparison using the more stringent depression threshold of ≥11 was not statistically significant (26.4% vs. 17.6%; *p* = 0.244). Similarly, anxiety scores >7 occurred in 62.3% versus 45.6% of patients (*p* = 0.068), whereas the proportion with anxiety scores ≥11 did not differ significantly (28.3% vs. 20.6%; *p* = 0.324). Given the exploratory nature and number of comparisons, the isolated *p* = 0.040 finding for depression >7 should be interpreted cautiously.

**Figure 2:**
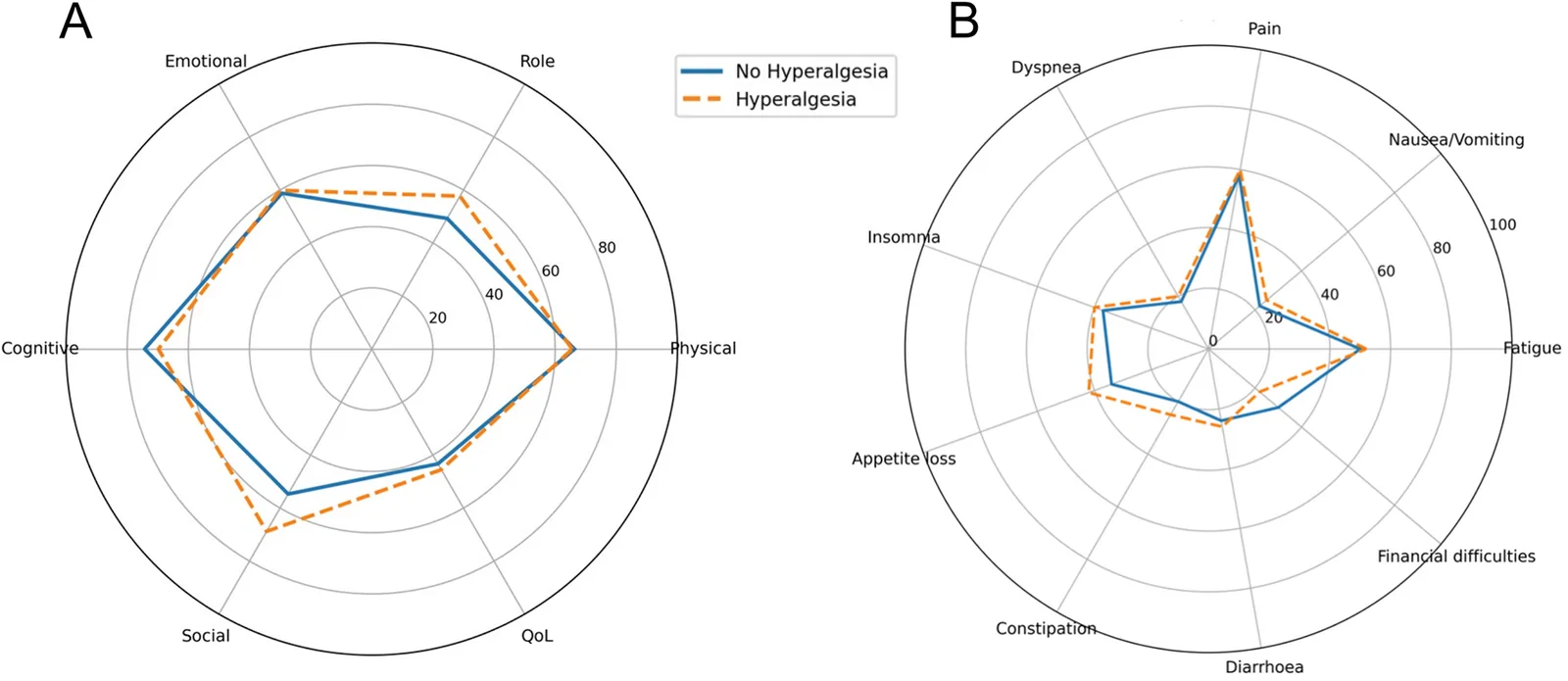
EORTC QLQ-C30 functional and symptom scales in patients with PDAC stratified by hyperalgesia. Radar plots showing EORTC QLQ-C30 functional **(A)** and symptom scales **(B)** in patients with PDAC (*n* = 121) stratified by hyperalgesia status (no hyperalgesia: *n* = 68, hyperalgesia: *n* = 53). Higher scores in **(A)** indicate better functioning. Higher scores in **(B)** indicate greater symptom burden. No statistically significant differences were observed between groups across all domains (Mann–Whitney U test; all *p* > 0.05).

The EORTC QLQ-C30 functional and symptom profiles according to hyperalgesia status are illustrated in Figure 2**.** No statistically significant differences were detected for any individual EORTC QLQ-C30 domain (all *p* > 0.05), although the descriptive profiles showed some variation between groups.

Exploratory analyses of patients with CP according to hyperalgesia status are presented in Supplementary Table S1.

The results of the exploratory disease-by-hyperalgesia interaction analyses are presented in Table 5. Models were adjusted for age, sex, and regular opioid use.

**Table 5:** Disease-by-hyperalgesia interaction analyses for selected patient-reported outcome measures.

PROM	*Interaction β*	*p-*value
BPI mean pain intensity	0.79 (95% CI −0.33–1.91)	0.164
BPI pain interference	1.61 (95% CI 0.27–2.95)	0.018
PCS total score	2.09 (95% CI −4.28–8.45)	0.519
Depression score	−0.36 (95% CI −2.55–1.84)	0.749
Anxiety score	−0.61 (95% CI −2.73–1.50)	0.569
EORTC QLQ-C30 global quality of life	−10.46 (95% CI −22.28–1.35)	0.082

Multivariable linear regression models included disease group, hyperalgesia status, the disease-by-hyperalgesia interaction term, age, sex, and regular opioid use. Interaction coefficients (β) with 95% confidence intervals (CI) are reported. CP and hyperalgesia served as the reference categories; therefore, the interaction term represents the differential association of normal versus hyperalgesic pain phenotype with the respective PROM in PDAC compared with CP.

A statistically significant disease-by-hyperalgesia interaction was only observed for BPI pain interference (interaction *β*=1.61, 95% CI 0.27 to 2.95; *p* = 0.018), providing evidence that the association between hyperalgesia status and pain interference differed between patients with CP and PDAC. No statistically significant disease-by-hyperalgesia interaction was detected for BPI mean pain intensity (*β*=0.79, 95% CI −0.33 to 1.91; *p* = 0.164), PCS total score (*β*=2.09, 95% CI −4.28 to 8.45; *p* = 0.519), depression score (*β*=−0.36, 95% CI −2.55 to 1.84; *p* = 0.749), anxiety score (*β*=−0.61, 95% CI −2.73 to 1.50; *p* = 0.569), or EORTC QLQ-C30 global quality of life (*β*=−10.46, 95% CI −22.28 to 1.35; *p* = 0.082).

## Discussion

This multicenter cross-sectional analysis compared pain processing and its impact on PROMs in prospectively enrolled cohorts of patients with painful CP and PDAC. The results demonstrated that patients with CP reported greater pain intensity, pain interference, pain catastrophizing, and financial difficulties than patients with PDAC. After adjustment for sex, age and regular opioid use, disease-by-hyperalgesia interaction analyses demonstrated evidence of a disease-specific association only for pain interference, whereas no clear interaction was detected for pain intensity, pain catastrophizing, depression, anxiety, or global quality of life.

### PROMs

In the presence of hyperalgesia, a deterioration in quality of life is to be expected compared to patients with normal pain processing. This has previously been demonstrated in CP patients and was also confirmed in the present study in this subgroup. In contrast to the findings in CP, no differences were observed in depression (*p* = 0.242)or anxiety scores (*p* = 0.126), pain catastrophizing (*p* = 0.257) or quality of life among PDAC patients with and without hyperalgesia. The higher prevalence of HADS depression scores >7 among patients with hyperalgesia was not accompanied by a difference in continuous depression scores or by a difference using the more stringent ≥11 threshold. Accordingly, the present data suggest that the clinical consequences of hyperalgesia in PDAC may be more selective than a general deterioration across psychological and quality-of-life domains.

Pain is the main symptom of CP and thus appears to be a key determinant of the quality of life (15). Patients with CP reported greater pain intensity (4.2 vs. 3.6, *p* = 0.027), a higher prevalence of moderate-to-severe pain (59% vs. 41%, *p* = 0.003), less pain relief (41.8 vs. 57.8, *p* < 0.001), and greater pain interference (4.1 vs. 3.4, *p* = 0.049) than patients with PDAC. In addition, pain catastrophizing was significantly more pronounced in CP, with higher PCS total scores compared with PDAC (23.3 vs. 20.1, *p* = 0.034). In PDAC, pain is one part of a whole symptom complex that also includes, for example, fatigue, nausea/ vomiting, loss of appetite, anorexia, weight loss, effects of tumor therapy and tumor progression (16). Pain, including hyperalgesia, therefore may not be as impactful in PDAC patients as in CP patients. In addition, a significant difference between the groups is the disease duration (74.4 vs. 1.7 months, *p* < 0.001). Given its short duration, pain is most probably not yet associated with reduced life quality. Instead, the recent tumor diagnosis, along with all its associated psychological aspects, takes precedence within the clinical symptomatology in that early period. The higher PCS scores and more pain on the BPI observed in CP in the present study are compatible with this concept and may reflect the longer duration and chronicity of pancreatic pain in this population.

Interestingly, the CP group reported significantly more financial difficulties (44.2 vs. 22.0, *p* < 0.001). This finding may be attributed to the longer disease course of CP patients, which results in long-term disability and an inability to work.

### Disease-specific association between hyperalgesia and PROMs

A particularly informative finding emerged from the formal disease-by-hyperalgesia interaction analyses. Within the CP cohort, hyperalgesia was associated with several adverse PROMs, including greater pain interference, greater pain catastrophizing, and lower global quality of life. A significant disease-by-hyperalgesia interaction was observed for BPI pain interference, indicating that the relationship between hyperalgesia and pain-related functional impairment differed between CP and PDAC after adjustment for age, sex, and regular opioid use.

The BPI interference construct captures the extent to which pain affects general activity, mood, walking ability, work, relationships, sleep, and enjoyment of life. In long-standing CP, persistent nociceptive input and altered central processing may progressively translate into impairment of everyday function. This interpretation is supported by longitudinal P-QST data showing that worsening hyperalgesia in recurrent acute and chronic pancreatitis can be accompanied by worsening pain interference and quality of life (17). In PDAC, the functional consequences of hyperalgesia may be modified by a substantially different disease trajectory. Patients are frequently confronted simultaneously with tumor-related symptoms, nutritional decline, psychological consequences of a cancer diagnosis, and progression-related or treatment-related morbidity. Thus, the same P-QST-defined sensory phenotype may coexist with a markedly different constellation of factors determining everyday function.

Importantly, the disease-specific effect was not universal across PROMs. Disease-by-hyperalgesia interactions were not statistically significant for pain intensity, pain catastrophizing, depression, anxiety, or global quality of life. The present results therefore point towards a selective disease-dependent relationship between altered pain processing and pain-related functional interference, rather than a general difference in the psychological or quality-of-life consequences of hyperalgesia.

### Pain processing

Pain in CP and PDAC arises in different pathological and clinical contexts. In PDAC, pain may result from tumor growth, perineural invasion, neuroimmune interactions, and infiltration of adjacent structures and neural plexuses (3, 13, 18, 19). In CP, pancreatic neuropathy likely plays a major role in many patients (20, 21), but other factors such as ductal obstruction accompanied by increased intraductal pressure and chronic inflammation may also mediate noxious stimuli (15). Despite these differences, both conditions can involve altered peripheral and central pain processing, providing a rationale for investigating P-QST-defined pain phenotypes across pancreatic diseases. A study comparing histological features demonstrated that neural density and neural hypertrophy are characteristic features of both PDAC and CP (18). This was associated with increased expression of GAP-43, a well-known marker for neuroplastic changes and increased tissue inflammation. In both conditions, neural hypertrophy was strongly associated with the severity of pain. The increase in neural density was associated with pain in CP, but not in PDAC patients.

The persistent hyperexcitability of spinal and supraspinal neurons causes peripheral and central sensitization and can thus contribute to the chronification of pain (12). This further leads to increased reactivity to somatic and visceral stimuli, which may clinically manifest as hyperalgesia and allodynia (11).

It should be noted that comparable pain processing was observed despite the inclusion of a significant number of PDAC patients with metastatic, i.e., extrapancreatic disease. This suggests that the local, pancreas-related pain stimulus, in particular, appears to play a role in the pathophysiology of altered central pain processing. This was also demonstrated in a previous analysis, in which hyperalgesia was found to be independent of the presence of metastases in PDAC (13).

P-QST was developed as a clinically feasible method to characterize pain processing beyond pain intensity alone and to categorize patients with CP into normal, segmental hyperalgesia, and widespread hyperalgesia phenotypes. Previous work demonstrated that widespread hyperalgesia in CP is associated with greater clinical pain and poorer quality of life, supporting the clinical relevance of altered pain processing in this population (5). More recently, abnormal central pain processing has also been demonstrated in patients with painful PDAC (13).

The present findings extend these observations by directly comparing patients with painful CP and PDAC. Hyperalgesia was present in 53.6% of patients with CP and 43.8% of evaluable patients with PDAC. After adjustment for age, sex, and regular opioid use, no statistically significant between-disease difference was detected in segmental or widespread hyperalgesia. Although this does not establish statistical equivalence, the frequent occurrence of hyperalgesia in both cohorts supports the concept that amplification of nociceptive processing is a relevant component of pancreatic pain across both inflammatory and malignant disease.

Among the individual P-QST measures, cold pressor endurance was longer in PDAC (79s vs. 74s, *β*=12,90 (95% CI: 1.24–24.57, *p* = 0.03), whereas conditioned pain modulation, pressure pain detection measures, and repetitive pinprick responses did not differ significantly between diseases. One possible explanation relates to the different duration of pain exposure in the two cohorts. Cold pressor endurance reflects sensitivity and tolerance to a remote noxious stimulus and has been associated with generalized pain sensitization in CP. Previous studies demonstrated shorter cold pressor endurance in sensitized compared with non-sensitized CP patients and in painful CP compared with healthy controls, with the latter finding being associated with increased spinal excitability (22, 23). The shorter endurance observed in CP in the present study may therefore reflect more established generalized pain sensitization resulting from prolonged nociceptive exposure, whereas most patients with PDAC were assessed relatively early in their disease and pain trajectory (5, 16).

### Clinical implications

The present findings suggest that pain phenotyping may also be clinically relevant in PDAC. Although P-QST-defined hyperalgesia has previously been characterized in CP and linked to clinical outcomes, our data indicate that similar sensory phenotypes are present in PDAC but may have different functional consequences.

For PDAC, P-QST could therefore provide additional information beyond pain intensity alone and help identify subgroups with distinct pain-processing patterns. Whether these phenotypes predict response to pharmacological or interventional pain treatments remains unknown and should be addressed in prospective PDAC-specific studies.

### Strengths and limitations

A major strength of this study is the large number of patients included in both groups, CP (153) and PDAC (122) which increases the robustness and statistical reliability of the findings. Furthermore, the multicenter design enhances the diversity of the study population and clinical setting. These factors improve the generalizability to the results and support their applicability to broader patient populations. The direct comparison of two distinct painful pancreatic diseases using the same standardized P-QST framework further strengthen the study (11, 12).

Nevertheless, there are some weaknesses. The cross-sectional design does not establish the temporal sequence between altered pain processing and clinical outcomes. This is relevant because central pain processing is not necessarily static. Previous work has demonstrated considerable neuroplasticity in chronic pancreatic pain (20), and recent longitudinal P-QST data indicate that individual pain phenotypes can change over time, with increasing hyperalgesia accompanying deterioration in pain interference and quality of life (17). Longitudinal assessment of PDAC will therefore be particularly important to determine whether pain processing evolves with increasing disease and pain duration.

Additionally, CP and PDAC cohorts differed substantially in age, racial composition, disease duration time, recruitment center, and medication exposure. Adjustment for age, sex and regular opioid use was applied consistently to the principal analyses, but residual confounding remains possible. The much shorter disease duration in the PDAC cohort could partially explain the observed differences and can only be addressed by analyzing longitudinal data from PDAC patients at a later stage of the disease.

In addition, a substantial proportion of patients with CP had a history previous or present heavy alcohol consumption. Alcohol exposure has been associated with neurobiological alterations contributing to both increased pain sensitivity and negative affective states, including anxiety and depression, suggesting that ongoing alcohol use may represent a potential confounder when interpreting pain phenotypes (24). Although acute alcohol intake may transiently reduce pain perception, chronic excessive alcohol consumption and withdrawal are associated with enhanced pain sensitivity, greater pain severity and adverse psychological outcomes (25).

Third, the PROM analyses were exploratory and included multiple outcomes. The principal interpretation should therefore focus on the overall pattern and the formal interaction. Finally, effective sample sizes varied between outcomes because of missing questionnaire and clinical data. Missing observations were not imputed, although missingness was limited for most measures.

## Conclusion

P-QST-defined hyperalgesia was frequent in patients with painful PDAC, highlighting altered pain processing as a relevant component of pancreatic cancer pain. Importantly, its clinical impact was not identical to that observed in CP. While hyperalgesia was not associated with a broad deterioration across PROMs in PDAC, the relationship between hyperalgesia and pain interference differed significantly between the two diseases.

These findings suggest that the clinical meaning of altered pain processing is disease-specific and cannot simply be extrapolated from CP to PDAC. Combining pain phenotyping with patient-reported outcomes may therefore provide a more comprehensive characterization of pancreatic cancer pain and offers a promising basis for future longitudinal and phenotype-guided treatment studies.

## Data Availability

The raw data supporting the conclusions of this article will be made available by the authors, without undue reservation.
