# Supplementary material for: Pain profiles in pancreatic cancer and chronic pancreatitis: a multicenter cross-sectional analysis
Source: Front Pain Res (Lausanne). 2026 Sep 11;7:1903789. doi: 10.3389/fpain.2026.1903789 (PMC13612469; doi:10.3389/fpain.2026.1903789)
Supplement: Supplementary file 1 [file Table1.docx]

**Supplemental Table 1.**

**Patient-reported outcome measures (PROMs) in patients with chronic pancreatitis stratified by hyperalgesia.**

|  | No hyperalgesia  (n = 71) | Hyperalgesia  (n = 82) | *p-values* |
| --- | --- | --- | --- |
| BPI mean  missings | 3 | 1 | 0.06^2^ |
| Mean (SD) | 3.8 (3) | 4.6 (3) |  |
| Opioid use  missings |  | 1 | 0.047^1^ |
| No | 34 (48%) | 26 (32%) |  |
| Yes | 37 (52%) | 55 (68%) |  |
| BPI relief  missings | 9 | 6 | 0.498^2^ |
| Mean (SD) | 40 (33) | 43.3 (29) |  |
| BPI severe  missings | 3 | 1 | 0.039^1^ |
| No | 34 (50%) | 27(33%) |  |
| Yes | 34 (50%) | 54 (67%) |  |
| BPI interference mean  missings | 3 | 1 | 0.024^2^ |
| Mean (SD) | 3.5 (3) | 4.6 (3) |  |
| PCS total  missings | 8 | 2 | 0.250^2^ |
| Mean (SD) | 20.4 (14) | 25.5 (13) |  |
| Depression score |  |  | 0.189^2^ |
| Mean (SD) | 6.3 (4) | 7.4 (4) |  |
| Depression score > 7  missings | 8 | 2 | 0.457^1^ |
| No | 37 (59%) | 42 (53%) |  |
| Yes | 26 (41%) | 38(47%) |  |
| Depression score **≥** 11  missings | 8 | 2 | 0.778^1^ |
| No | 50 (79%) | 65 (81%) |  |
| Yes | 13 (21%) | 15 (19%) |  |
| Anxiety score  missings | 8 | 2 | 0.25^2^ |
| Mean (SD) | 7.7 (5) | 8.4 (4) |  |
| Anxiety score > 7  missings | 8 | 2 | 0.225^1^ |
| No | 34 (54%) | 35 (44%) |  |
| Yes | 29 (46%) | 45 (56%) |  |
| Anxiety score **≥** 11  missings | 8 | 2 | 0.507^1^ |
| No | 48 (76%) | 57 (71%) |  |
| Yes | 15 (24%) | 23 (29%) |  |
| EORTC physical functioning  missings | 1 | 1 | 0.401^2^ |
| Mean (SD) | 74.4 (18) | 70.3 (23) |  |
| EORTC role functioning  missings | 1 | 1 | 0.04^2^ |
| Mean (SD) | 58.6 (31) | 47.1 (35) |  |
| EORTC emotional functioning  missings | 1 | 0 | 0.546^2^ |
| Mean (SD) | 62.3 (30) | 61.6 (26) |  |
| EORTC cognitive functioning  missings | 1 | 0 | 0.13^2^ |
| Mean (SD) | 71.2 (27) | 64.0 (30) |  |
| EORTC social functioning  missings | 1 | 2 | 0.061^2^ |
| Mean (SD) | 63.1 (32) | 52.9 (34) |  |
| EORTC quality of life  missings | 1 | 3 | 0.014^2^ |
| Mean (SD) | 51.7 (23) | 41.8 (26) |  |
| EORTC general symptoms  missings | 1 | 2 | 0.04^2^ |
| Mean (SD) | 38.3 (20) | 44.9 (23) |  |
| EORTC fatique  missings | 1 |  | 0.288^2^ |
| Mean (SD) | 51.1 (27) | 55.2 (27) |  |
| EORTC fatique  missings | 1 |  | 0.013^2^ |
| Mean (SD) | 22.4 (27) | 35.4 (33) |  |
| EORTC fatique  missings | 1 |  | 0.051^2^ |
| Mean (SD) | 55.7 (31) | 64.8 (34) |  |
| EORTC fatique  missings | 1 |  | 0.569^2^ |
| Mean (SD) | 18.1 (28) | 20.7 (29) |  |
| EORTC fatique  missings | 1 |  | 0.465^2^ |
| Mean (SD) | 48.1 (37) | 52.8 (40) |  |
| EORTC fatique  missings | 1 |  | 0.153^2^ |
| Mean (SD) | 38.6 (31) | 47.2 (37) |  |
| EORTC fatique  missings | 1 |  | 0.337^2^ |
| Mean (SD) | 23.3 (29) | 20.7 (30) |  |
| EORTC fatique  missings | 1 |  | 0.117^2^ |
| Mean (SD) | 21.9 (27) | 31.3 (34) |  |
| EORTC fatique  missings | 1 | 2 | 0.072^2^ |
| Mean (SD) | 38.1 (39) | 49.6 (39) |  |

Data are presented as mean ± SD or n (%). Categorial variables were compared using the χ² test^1^ and continuous variables using the Mann–Whitney U test^2^. Depression and anxiety are shown as continuous scores and by established cut-offs (>7 and >11). BPI mean represents average pain intensity (0–10). BPI severe pain (mean 4–10) indicates patients with a mean pain score ≥4 (moderate to severe pain). BPI relief reflects percentage pain reduction, and pain interference describes pain-related functional impairment. EORTC QLQ-C30 scales range from 0–100; higher scores indicate better functioning (function scales) or greater symptom burden (symptom scales). Abbreviations: CP, chronic pancreatitis; PDAC, pancreatic ductal adenocarcinoma; SD, standard deviation; BPI, brief pain inventory; PCS, pain catastrophizing scale.
